# Broad-Spectrum Coronavirus Fusion Inhibitors to Combat COVID-19 and Other Emerging Coronavirus Diseases

**DOI:** 10.3390/ijms21113843

**Published:** 2020-05-28

**Authors:** Xinling Wang, Shuai Xia, Qian Wang, Wei Xu, Weihua Li, Lu Lu, Shibo Jiang

**Affiliations:** 1 Key Laboratory of Medical Molecular Virology (MOE/NHC/CAMS), School of Basic Medical Sciences, Fudan University, Shanghai 200032, China; 18111010064@fudan.edu.cn (X.W.); 15111010053@fudan.edu.cn (S.X.); Wang_qian@fudan.edu.cn (Q.W.); Xuwei0576@126.com (W.X.); 2 Key Laboratory of Reproduction Regulation of National Health Commission, (Shanghai Institute of Planned Parenthood Research), Fudan University, Shanghai 200032, China; weihua.li@sippr.org.cn

**Keywords:** COVID-19, peptide, antibody, fusion inhibitor, entry inhibitor, protease inhibitor

## Abstract

In the past 17 years, three novel coronaviruses have caused severe acute respiratory syndrome (SARS), Middle East respiratory syndrome (MERS), and the coronavirus disease 2019 (COVID-19). As emerging infectious diseases, they were characterized by their novel pathogens and transmissibility without available clinical drugs or vaccines. This is especially true for the newly identified COVID-19 caused by SARS coronavirus 2 (SARS-CoV-2) for which, to date, no specific antiviral drugs or vaccines have been approved. Similar to SARS and MERS, the lag time in the development of therapeutics is likely to take months to years. These facts call for the development of broad-spectrum anti-coronavirus drugs targeting a conserved target site. This review will systematically describe potential broad-spectrum coronavirus fusion inhibitors, including antibodies, protease inhibitors, and peptide fusion inhibitors, along with a discussion of their advantages and disadvantages.

## 1. Introduction

The pandemic of coronavirus disease (COVID-19) was caused by the novel coronavirus 2019 (2019-nCoV) [1], also known as human coronavirus 2019 (HCoV-19) [2] or severe acute respiratory syndrome coronavirus 2 (SARS-CoV-2) [3]. It has posed a serious threat to global public health, as well as social and economic stability, thus calling for the development of highly effective therapeutics and prophylactics [4].

In its research and development blueprint, the World Health Organization (WHO) announced the first list of prioritized diseases in 2015 and formally announced it on 9 February 2018 [5]. Apart from SARS and MERS, Disease X has sparked an international epidemic caused by an unknown pathogen that would be highly transmissible among humans. Jiang et al. suggested that the first reported pneumonia cluster in Wuhan in December of 2019, with its etiology unknown (later known as 2019-nCoV), should be recognized as the first Disease X [6]. However, this was the third coronavirus that has caused severe pneumonia in humans over the past twenty years. SARS-CoV infection resulted in 8096 cases and 774 deaths [7], whereas confirmed MERS cases numbered about 2494, including 858 deaths [8]. As of 25 April 2020, 2,724,809 COVID-19 cases and 187,847 deaths were confirmed [9], with no signs of abatement. As noted previously, vaccines and FDA-approved drugs still remain out of clinical reach. These facts call for the development of broad-spectrum anti-coronavirus drugs targeting a conserved target site that would address the current urgency and those coronavirus outbreaks that are likely to emerge in the future.

**Figure 1:**
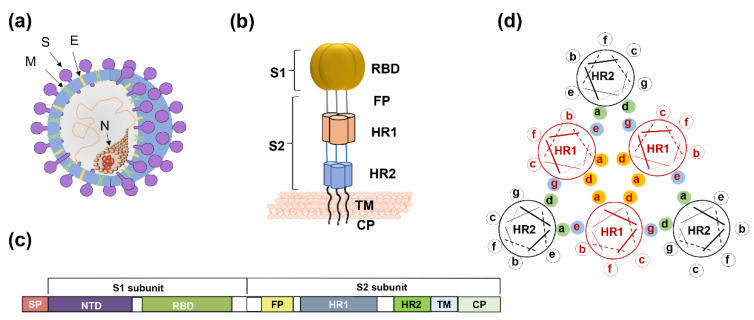
The spike protein of coronavirus and model of membrane fusion mechanism. (**a**) Cartoon figure of coronavirus structural protein. Three transmembrane proteins, spike protein (S; purple), membrane protein (M; green), envelop protein (E; yellow) are found on the surface of the coronavirus envelope. The nucleocapsid protein (N; orange) encapsulates the viral genome inside the virion. (**b**) Structure of the Spike protein. S protein contains two subunits, S1 and S2. S1 includes the receptor-binding domain (RBD; dark yellow). S2 includes the HR1 region (light orange) and the HR2 region (light blue). (**c**) The genomic region of complete coronavirus. (**d**) Interaction between HR1 and HR2. Residues located at the “a” and “d” positions in HR1 helices (shown as yellow circle shadow) interact to form an internal trimer; residues at “e” and “g” positions (blue shadow) interact with the residues at the “a” and “d” positions (green shadow) in the HR2 helices to form 6-HB.

Coronaviruses (CoVs) comprise four genera—alphacoronavirus, betacoronavirus, gammacoronavirus, and deltacoronavirus. SARS-CoV-2 belongs to β-coronavirus. In this group, highly pathogenic SARS-CoV and MERS-CoV caused severe human diseases in 2002 and 2012, respectively [10,11]. The genome sequence of SARS-CoV-2 is 79.5% homologous to SARS-CoV and 96% identical to bat SARS-related coronavirus (SARSr-CoV) [12,13]. Four low-pathogenicity coronaviruses are also epidemic in humans—HCoV-NL63, HCoV-229E, HCoV-OC43, and HCoV-HKU1. The viral genome encodes four structural proteins, spike protein (S), membrane protein (M), envelope protein (E), and nucleocapsid protein (N) (Figure 1a). The S protein is a type I transmembrane glycoprotein, and it includes an extracellular domain, transmembrane domain, and intracellular domain. The extracellular domain of the S protein contains two subunits, S1 and S2, each playing a different role in receptor recognition, binding, and membrane fusion (Figure 1b). The S1 subunit includes the N-terminal domain (NTD) and C-terminal domain (CTD). Generally, the receptor-binding domain (RBD) is located in the CTD (Figure 1c). NTD mediates the binding between the virus and sugar-based receptors, and the CTD mediates viral binding to the protein-based receptor [14]. SARS-CoV, SARS-CoV-2, and MERS-CoV utilize the CTD to bind their respective receptors. Receptor recognition and binding trigger membrane fusion between the virus and the target cell. We expect some conserved sites to be involved in these processes, in view of the fact that membrane fusion is an essential step for coronavirus infection of target cells. Taking membrane fusion as our focal point, this review will systematically describe broad-spectrum coronavirus fusion inhibitors, along with a discussion of their advantages and disadvantages.

## 2. The Mechanism of Membrane Fusion

### 2.1. Receptor Recognition and Binding

Receptor recognition by the S1 subunit of the spike protein of coronaviruses is the first step of viral infection [15], followed by RBD binding to the receptors. SARS-CoV-2 and SARS-CoV use angiotensin-converting enzyme 2 (ACE2) as a receptor to mediate viral entry into target cells [12]. A study reported that the affinity of the ectodomain of SARS-CoV-2 S protein to ACE2 is 10- to 20-fold higher than that of SARS-CoV S protein [16]. However, another team revealed a similar ACE2 binding affinity between SARS-CoV-2 and SARS-CoV [17]. During the SARS-CoV infection, its spike protein could induce the down-regulation of ACE2 [18], while SARS-CoV-2 might share a similar mechanism to regulate ACE2 expression. Additionally, in COVID-19 patients, not all ACE2-expressing organs had similar level of pathophysiology, implying that some other mechanisms might also mediate the tissue damage; thus, further studies on the interaction between SARS-CoV-2 and ACE2 are warranted [19]. ACE2 is also the receptor of HCoV-NL63 [20]. HCoV-229E uses aminopeptidase N (APN) for cell entry [21], whereas MERS-CoV utilizes dipeptidyl peptidase 4 (DPP4, CD26) as its receptor [22], indicating the diversity of receptors among human coronaviruses. It is the interaction between RBD and the receptor that determines viral infection spectrum and host range [23]. Among the different genera, coronaviruses share low similarity in RBD sequence [24], which might explain the use of different receptors. The amino acid sequence in RBD of SARS-CoV-2 shares ~73% similarity with SARS-CoV and SARSr-CoVs (e.g., WIV1 and Rs3367), respectively, while it only has a 21% similarity with that of MERS-CoV [25]. Importantly, the RBD contains several neutralization epitopes that can serve as a target for the development of vaccines and antibodies [26,27,28,29]. At the same time, variation in the RBD limits the broad-spectrum property of vaccines and antibodies. Nonetheless, it is still possible to utilize the relatively conserved neutralization epitopes among the SARS-CoV and SARS-CoV-2, for research and development of broad-spectrum neutralizing antibodies, against lineage B β-CoVs, including SARS-CoV-2, SARS-CoV, and SARSr-CoVs.

### 2.2. Proteolytic Function in Membrane Fusion

In the natural state, S protein on the surface of coronavirus is inactive. Only after receptor binding and S protein priming by proteolysis of proteases is the S protein activated and fusion triggered [30]. The S2 subunit is then exposed to mediate membrane fusion. In all coronaviruses, a site called S2′ that is located upstream of the fusion peptide (FP) of the S protein, can be cleaved by the host protease [31,32]. At the boundary of the S1/S2 subunits, a study found that SARS-CoV-2 S protein contains a furin cleavage site that is cleaved during biosynthesis, which is different from SARS-CoV and SARSr-CoVs [17] but is similar to MERS-CoV [33]. The virus can enter into the target cell through two routes—direct fusion on the cellular surface and endocytosis. For the endocytosis route, the virus is encapsulated by the endosome after receptor binding. Then, the low pH environment promotes the cleavage of the S protein with pH-dependent cysteine protease cathepsin L (CPL). For direct fusion on the cellular membrane, transmembrane protease serine 2 (TMPRSS2) plays roles in cleavage and activation [34]. Some studies reported that SARS-CoV S protein could also be cleaved with human airway trypsin-like protease [34,35,36]. TMPRSS2 can also promote MERS-CoV entry into the target cell through the endocytosis pathway [37]. A recent study demonstrated that SARS-CoV-2 utilizes TMPRSS2 to activate the S protein and that a protease inhibitor could inhibit pseudovirus entry [38]. These studies suggest that coronaviruses have a similar proteolytic process and use the same protease, such as TMPRSS2. Therefore, suppressing the proteolysis of proteases might be a path toward the development of broad-spectrum fusion inhibitors.

### 2.3. Mechanism of S2 Subunit-Mediated Membrane Fusion

For coronaviruses, the S protein binding of receptor initially activates the entry process, resulting in membrane fusion and ensuring virus infection of the target cell. This mechanism was confirmed in SARS-CoV and MERS-CoV [39,40]. After RBD binds to cellular receptors, the conformation of S2 subunits changes, followed by exposure of the fusion peptide into the cell membrane. S2 also includes the HR1 and HR2 regions that interact. Residues located at the “a” and “d” positions in the HR1 helices interact to form an internal trimer, and residues at the “e” and “g” positions interact with the residues at the “a” and “d” positions in the HR2 helices to form a six-helix bundle (6-HB) (Figure 1d). The 6-HB helps the viral membrane and cell membrane to come into close contact for viral fusion and entry.

In native conformation, the S2 subunit is buried inside the S1 subunit and only when fusion occurs, the S2 subunit is instantly exposed. Through sequence alignment, Xia et al. found that the S2 subunit of SARS-CoV-2 is highly conserved, in which the HR1 and HR2 domains share a 92.6% and 100% identity with those of SARS-CoV, respectively [41]. They designed two peptides derived from the HR1 and HR2 domains, 2019-nCoV-HR1P and 2019-nCoV-HR2P, respectively, and confirmed that they could interact with each other to form the coiled-coil complex, as shown by using native electrophoresis and circular dichroism. Through crystallographic analysis, the parallel trimeric coiled-coiled center formed by three HR1 domains was surrounded by three HR2 domains, in an antiparallel manner [34]. Thus, SARS-CoV-2 enters the target cell through membrane fusion in a 6-HB-dependent manner.

Therefore, coronaviruses appear to have a similar mechanism of membrane fusion, while HR1 and HR2 regions are highly conserved and can serve as important targets for development of broad-spectrum coronavirus fusion inhibitor-based drugs, for the treatment and prevention of coronavirus diseases.

## 3. Broad-Spectrum Coronavirus Fusion Inhibitors

**Figure 2:**
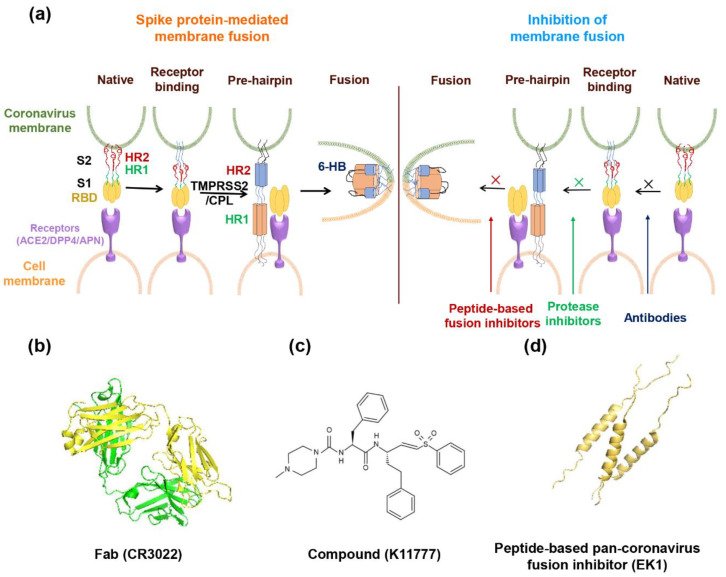
Model of spike protein (S)-mediated membrane fusion and mechanism of inhibition of membrane fusion with drugs. (**a**) In the native state, the S1 subunit encapsulates the S2 subunit. After receptor binding, the S2 subunit is exposed, and the fusion peptide inserts into the target cell membrane. Then, 3-HR1 interacts with 3-HR2 to form the 6-HB structure, followed by membrane fusion. Antibodies block the receptor recognition. Protease inhibitors against TMPRSS2/CPL shuts down S protein priming. Peptide fusion inhibitor binds to the HR1 region, inhibiting the formation of 6-HB. (**b**–**d**) The structures of a representative neutralizing antibody (IgG Fab of CR3022), a small molecule inhibitory compound (K11777) and a peptide-based pan-coronavirus fusion inhibitor (EK1).

In this section, we summarize the broad-spectrum coronavirus fusion inhibitors targeting RBD in the S1 subunit of coronavirus S protein, the fusion-related proteases, and the HR1 domain in the S2 subunit of coronavirus S protein, respectively, under development (Figure 2).

### 3.1. Broad-Spectrum Coronavirus Fusion Inhibitors Targeting RBD

Fusion inhibitors include antibodies, small molecules, and peptide inhibitors [39,40,42,43,44,45]. In this part, we review antibodies and small molecules with broad-spectrum activity targeting RBD in the S1 subunit of coronavirus S protein.

Several specific antibodies targeting S protein for coronavirus were reported, such as SARS-CoV neutralizing antibodies CR3022, m396, and S109.8 [46,47], MERS-CoV neutralizing antibodies m336 [48] and SAB-301 [48,49], and SARS-CoV-2 antibodies 31B5 and 32D4 [50], but no broad-spectrum coronavirus antibody was reported. Some antibodies were shown to have cross-reactivity among some coronaviruses [51,52]. One study demonstrated that a SARS-CoV-specific monoclonal antibody (mAb), CR3022, showed cross-reactive binding to SARS-CoV-2 RBD, but its targeting epitope did not overlap the ACE2 binding site [53]. Sera from convalescent SARS patients showed cross-activity in blocking SARS-CoV-2 pseudovirus entry [38]. Recombinant ACE2-Ig fusion protein exhibited neutralizing activity on pseudotyped SARS-CoV-2 and SARS-CoV, both of which utilized ACE2 as their receptor, with inhibitory concentration (IC_50_) values of 0.1 and 0.8 μg/mL, respectively (Table 1). It inhibited SARS-CoV-2 and SARS-CoV S-mediated cell–cell fusion with the IC_50_ of 0.65 and 0.85 μg/mL, respectively [54]. Some studies showed that ACE2 negatively regulated the renin-angiotensin system (RAS), and ACE2 and the angiotensin II receptor (AT2) could reduce mouse lung damage induced by sepsis or acid aspiration [55]. Coronavirus down-regulated ACE2 and resveratrol (RES), which experimentally deactivated the RAS system, resulting in an increase of ACE2 [56]. These studies revealed that more attention should be paid to the role of ACE2 in coronavirus infection in the development of putative therapeutic measures. Another study showed that SARS-CoV RBD protein-immunized mouse serum cross-neutralized with SARS-CoV-2, but that MERS-CoV RBD-specific polyclonal antibody had no cross-reactivity [52]. A recent study reported a cross-neutralizing human antibody, 47D11, which could bind to full-length spike protein expressed on cells (Table 1). The antibody targets a conserved epitope in the RBD region. It could inhibit pseudotyped SARS-CoV and SARS-CoV-2 infection of Vero E6 cells with the IC_50_ values of 0.06 and 0.08 μg/mL, respectively. 47D11 could also inhibit live SARS-CoV and SARS-CoV-2 infection with the IC_50_ of 0.19 and 0.57 μg/mL, respectively, through the plaque reduction neutralization test (PRNT) assay. At a concentration of 20 μg/mL, the cell–cell fusion mediated by SARS-CoV-S and SARS-CoV-2 S could be inhibited, whereas it could not inhibit fusion mediated by MERS-CoV S protein. However, a mechanism that is not dependent on receptor binding interference, remains unknown [51]. Pseudotyped SARS-CoV- and MERS-CoV-immunized mouse serum was demonstrated to have no inhibitory activity against SARS-CoV-2 infection [25]. The lack of efficacy for these MERS-CoV antibodies could be attributed to the difference in RBD and receptors between SARS-CoV and SARS-CoV-2. No cross-antibodies against MERS-CoV, SARS-COV, and SARS-CoV-2 were found. This revealed the shortcomings in antibodies compared to peptides in achieving broad-spectrum coronavirus cross-neutralizing activity.

Some small molecules also showed antiviral activity, such as ACE2 inhibitors [43] and interferon-inducible transmembrane (IFITM) proteins [57]. N-(2-aminoethyl)-1 aziridine-ethanamine, a novel ACE2 inhibitor, could inhibit the activity of ACE2 with an IC_50_ value of 57 ± 7 μM, and could block the SARS-CoV S protein-mediated membrane fusion, with a concentration in the micromolar range [43]. However, it might only have efficacy on coronavirus using ACE2 as a receptor and, hence, is difficult to achieve the goal of a broad-spectrum anti-coronavirus drug. IFITM can inhibit host cell entry of several enveloped viruses by promoting the accumulation of cholesterol in endosomes. It could inhibit the entry of MERS-CoV, SARS-CoV, HCoV-229E, and HCoV-NL63, but it is not a specific inhibitor for coronavirus [57]. Thus, small molecules that block receptor binding need further research.

### 3.2. Fusion Inhibitors Targeting Fusion-Related Proteases

At present, research on protease inhibitors mainly focuses on small molecules. Recent studies reported that protease inhibitors could block SARS-CoV-2 entry by inhibiting TMPRSS2 [38]. Some studies detected the ability of protease inhibitors to prevent SARS-CoV-2 infection of cells (Table 2). Camostat mesylate, a clinical drug used for chronic pancreatitis in Japan, could suppress TMPRSS2 activity. It could inhibit Calu-3 cell infection with pseudotyped SARS-CoV-2 with the EC_50_ (concentration for 50% of maximal effect) value of about 1 μM and EC_90_ (concentration for 50% of maximal effect) value of about 5 μM [38]. At a concentration of 50 μM, it could inhibit 80% pseudotyped SARS-CoV-2 entry into primary human airway epithelial cells [38]. Camostat mesylate also showed inhibitory activity on SARS-CoV-S- and MERS-CoV-S-mediated entry [38,58,59]. Nafamostat mesylate (NM), consistent with camostat mesylate, could inhibit SARS-CoV-2 infection of calu-3 cells [60]. In another study, the EC_50_ of camostat mesylate against SARS-CoV-2 S-mediated entry into calu-3 cells was 87 nM. The activity of nafamostat mesylate was about 15-fold greater than that of camostat mesylate, with EC_50_ of 5 nM. Moreover, it could inhibit TMPRSS2-dependent host cell entry of pseudotyped SARS-COV and MERS-CoV, with IC_50_ values of 1.4 and 5.9 nM, respectively [60]. In addition, nafamostat mesylate could inhibit MERS-CoV S-mediated membrane fusion with an IC_50_ value of 100 nM, whereas the IC_50_ of camostat mesylate was 1000 nM [61]. NM was more efficient in reducing MERS-CoV RNA internalization at a concentration of 1 nM [61]. However, gabexate mesylate (FOY), another protease inhibitor, only slightly inhibited SARS-CoV- and SARS-CoV-2 S-mediated entry into calu-3 cells, with EC_50_ of 115 μM and 1.2 M, respectively, but had no effect on MERS-CoV infection [60].

Besides the TMPRSS2 inhibitors, it was reported that K11777, a small molecule compound-based cathepsin-L like protease inhibitor [62] could inhibit infection of the pseudotyped SARS-CoV, MERS-CoV, HCoV-229E, and HCoV-NL63, with the IC_50_ values of 0.68, 46.12, 1.48, and 6.78 nM, respectively, and could block live SARS-CoV replication in Vero 76 cells. However, K11777 could fully inhibit virus entry only in cells lacking activating serine protease. Otherwise, K11777 should combine with serine protease inhibitor, for example, camostat and nafamostat. The derivates of K11777, SMDC256159, and SMDC256160, had similar antiviral effects in vitro. In SARS-CoV lethal infection mouse model, single usage of SMDC256160 exhibited no significant protection, while camostat could protect about 60% mice from death [63]. The in vivo efficiency of these protease inhibitors have not yet been reported. Another cathepsin L inhibitor, SID-26681509 at 2 μM could inhibit 76% SARS-CoV-2 S pseudovirions for entry into 293/hACE2 cells [64]. Teicoplanin, a glycopeptide antibiotic, could potently inhibit CPL in the late endosome/lysosome; thus, blocking the entry of coronavirus. It inhibited pseudotyped MERS-CoV and SARS-CoV entry with the IC_50_ values of 630 and 3760 nM, respectively [65]. Recently, a study showed that teicoplanin inhibits pseudotyped SARS-CoV-2 entry with an IC_50_ value of 1660 nM. Other glycopeptide antibiotics, dalbavancin, also exhibited inhibitory activity, but vancomycin did not block the entry of SARS-CoV-2 [66]. Additionally, a broad-spectrum cysteine protease inhibitor E64D at 30 μM could inhibit 92.5% SARS-CoV-2 pseudovirions for entry [64]. However, the 50% cell cytotoxicity concentration (CC_50_) of some small molecules were never reported. To evaluate whether the small molecules could be developed as a broad-spectrum coronavirus fusion-inhibitor-based drug, its safety and bioactivity in vivo needs to be assessed.

### 3.3. Broad-Spectrum Coronavirus Fusion Inhibitors Targeting the HR1 Domain

Currently, research on fusion inhibitors is mainly focused on peptide drugs. Researchers have discovered many peptides that can inhibit coronavirus infections, such as CP1 derived from SARS-CoV HR2 [39], MERS-HR2P derived from MERS-CoV HR2 [40], and 2019-nCoV-HR2P derived from SARS-CoV-2 HR2 [41]. Recently, we reported some pan-coronavirus (pan-CoV) peptide fusion inhibitors. These peptide fusion inhibitors, including OC43-HR2P, EK1, and EK1C4 [34,44], inhibit human coronavirus fusion by targeting the HR1 domain, as shown in Table 1. OC43-HR2P could inhibit MERS-CoV-, SARS-CoV-, and HCoV-OC43 S-mediated cell–cell fusion with IC_50_ values of 0.39, 0.54, and 0.66 μM, respectively. It also showed an effective inhibitory activity against 229E- and NL63 S-mediated cell–cell fusion with IC_50_ of 0.84 and 0.94 μM, respectively [44]. By introducing the negatively and positively charged amino acids Glu (E) and Lys (K), the new peptide EK1 was obtained. EK1 peptide also targeted the HR1 domain and shared a mechanism of action in common with other peptide fusion inhibitors, including SJ-2176 [67], CP-1 [39], and MERS-HR2P [40]. EK1 blocks the fusion of viral and target cell membranes and entry into the target cell by competitively inhibiting the formation of 6-HB. Extensive hydrophobic interactions in EK1 and hCoVs HR1 guaranteed the broad-spectrum neutralization of EK1. EK1 peptide had broad-spectrum antiviral activity against five human coronaviruses (pseudotyped SARS-CoV, MERS-CoV, 229E, OC43, and NL63) and three bat-SARSr-CoVs (pseudotyped Rs3367, WIV1, SHC014) in vitro, with IC_50_ values ranging from 0.26 to 6.02 μM. Moreover, the pan-CoVs fusion inhibitor EK1 also had a preventive and protective effect in vivo. It could protect hDPP4-transgenic mice, or regular mice, from HCoV-OC43 or MERS-CoV infection via the intranasal route [44]. The structural basis of broad-spectrum inhibitory activity is that EK1 can snugly insert into the hydrophobic groove formed by HR1 and then deliver a 6-HB architecture similar to that of 3HR1-3HR2 6-HB, thus blocking virus infection. The hydrophobic, chain-to-side chain hydrophilic interactions and ridge-packing interactions between the EK1 and 3HR1 cores ensure the pan-CoV’s inhibitory activity [44].

Although the HR1 region of 2019-nCoV has about a 38% difference from that of SARS-CoV, the EK1 peptide could still bind to the 2019-nCoV-HR1P, inhibit S-mediated cell–cell fusion with IC_50_ of 0.19 μM, and could significantly inhibit pseudotyped SARS-CoV-2 entry with an IC_50_ value of 2.38 μM [41].

EK1 peptide was conjugated with cholesterol at its C-terminus. The resultant lipopeptide EK1C4 exhibited significantly enhanced anti-CoV activity, about 150- and 240-fold over that of EK1 peptide against pseudotyped SARS-CoV-2 infection and S-mediated membrane fusion [34]. Most importantly, EK1C4 could inhibit infection of live HCoVs, including SARS-CoV-2, MERS-CoV, HCoV-NL63, HCoV-229E, and HCoV-OC43, with IC_50_ values ranging from 4 to 188 nM (Table 3). To evaluate the potential prophylactic effect, the authors utilized EK1C4 to treat HCoV-OC43- infected newborn mice, before or after infection. They found that the mice treated with EK1C4 at 0.5, 2, and 4 h before the virus infection all lived, while mortality of mice in the pre-2-h group treated with the EK1 peptide was similar to the viral control group [34]. These results suggested that the antiviral activity of EK1C4 was higher than that of the EK1 peptide. Results from the experiments with both EK1 and EK1C4 highlighted that the peptide fusion inhibitors did have the potential for development as promising antiviral agents.

## 4. Advantages and Disadvantages of the Broad-Spectrum Coronavirus Fusion Inhibitors

Broad-spectrum coronavirus fusion inhibitors have different strengths and weaknesses, and we summarize them in this part.

The main advantages of a human neutralizing antibody include its high safety, stability, and efficacy. However, the highly conserved HR1 region is not a good target for antibody IgG with a molecular weight of 150 kD, because a protein with more than 71 kD cannot easily access the HR1-trimer in the fusion-intermediate stage [68,69]. Consequently, antibodies for coronavirus usually target the RBD domain or the S1 subunit, not the HR1 region. RBD is usually considered a promising target for developing specific antibodies [48]. However, there are no reported broad-spectrum neutralizing antibodies against divergent coronaviruses. As described above, mouse antiserum or monoclonal antibody against SASRS-CoV showed cross-reactivity in SARS-CoV-2, and SARSr-CoVs [25,51,52]. Thus, it is still possible to develop broad-spectrum neutralizing antibodies against lineage B betacoronaviruses. However, there are also some disadvantages to antibodies. First, the high cost of antibodies increase the burden on patients. Additionally, treatment with a single antibody could induce escape mutations in RBD, for instance, the MERS-COV antibody CDC2-C2 [70]. Moreover, the RBD is a high mutable region of the coronavirus S protein. As reported in a recent study, mAbs with an epitope of SARS-CoV RBD did not recognize the SARS-CoV-2 RBD region and showed low cross-reactivity to it [16].

Small molecule fusion inhibitors targeting serine protease have some advantages over antibodies. First, they can be administered orally. Second, they can be stored and transported at normal temperature. Third, they are economical to synthesize and easily acceptable by patients. However, proteases are widely present in various tissues and cells; consequently, protease-targeting inhibitors could have side effects and produce toxic responses. One study reported that camostat mesylate caused acute eosinophilic pneumonia [71]. Nafamostat mesylate has a short half-life with risk of bleeding [72]. Moreover, the single-use of SMDC256160 had no protective effect in vivo. Thus, whether protease inhibitor could effectively inhibit coronaviruses infection in vivo still requires more research work.

Pan-CoV peptide fusion inhibitors have numerous advantages. First, the EK1 peptide has low immunogenicity with no detectable EK1-specific antibodies in EK1-immunized mouse serum, over a two-week period [44]. Second, the targeting site of the EK1 peptide is the HR1 domain in the S2 subunit. This region, as noted above, is highly conserved, thus guaranteeing the inhibitory activity of pan-CoVs. Third, since the S2 subunit is instantly exposed, drug resistance is not easily induced. Fourth, EK1 has excellent druggable properties, has good solubility in water, and shows superior stability. Compared to the EK1 peptide, EK1C4 has a more potent inhibitory activity. It can treat HCoV-OC43-infected mice and protect mice from virus infections via the intranasal route [34,44]. It can also be administered to patients via inhalation. Moreover, local application is expected to be much safer than systemic administration [34,44]. Generally, peptide drugs are safer than chemical medications because peptide drugs have good specificity and biological activity, a clear mechanism of drug action, and often have fewer side effects [34,44,73]. Therefore, lipopeptide EK1C4 is a promising candidate for development as a pan-CoV fusion inhibitor-based therapeutic and prophylactic for the treatment and prevention of the current COVID-19 and MERS, and future emerging and reemerging coronavirus diseases. EK1C4 can be inhaled as an aerosol formulation to reduce the viral load in the lung, thus alleviating the pulmonary inflammatory reaction and reducing the chance of transmission to high-risk groups. An intranasal formulation of EK1C4 can prevent infection [34]. Different from small molecules, peptide drugs do indeed represent a unique class. For example, peptides have been developed as drugs for infectious disease and cancer treatment, such as the anti-HIV peptide drug T20 approved by the FDA to treat AIDS [74]. Of course, peptide drugs have disadvantages. First, most peptide drugs can only be synthesized, increasing the cost, compared to small molecules. Second, peptide drugs might be more expensive than smaller molecule compound drugs because of the high cost of peptide production. Third, peptide drugs generally have a shorter half-life than antibody drugs [75]. Finally, peptide drugs cannot be taken orally, making them less convenient than small molecules.

## 5. Summary

The entry processes of a coronavirus include receptor recognition and binding, proteolytic cleavage of S protein, and S2 subunit-mediated membrane fusion. Each of these processes can serve as a target for the development of broad-spectrum drugs. Neutralizing antibodies that generally target the RBD in S protein have the advantages of high safety, stability, and efficacy; but the high variation of the RBD region among coronaviruses limits the development of broad-spectrum neutralizing antibodies. The high cost of production is another negative factor for their clinical applications. The small molecule compound-based entry inhibitors can be taken orally, with high acceptance. However, they are generatelly more toxic and less effective than antibodies and peptides in blocking virus entry through protein-protein interaction. Since many viruses use the proteases to mediate membrane fusion, they can be used as targets for development of broad-spectrum virus fusion inhibitors. However, they are human proteins and have their own functions in human bodies. Therefore, application of an inhibitor of the related enzyme might cause some side-effects. Peptide-based pan-CoV fusion inhibitors have shown great promise in the development of broad-spectrum prophylatics or therapeutics for the prevention and treatment of the current COVID-19, MERS, and other coronavirus diseases, as well as the emerging and reemerging coronavirus diseases that will occur in the future. Their target site, the HR1-trimer at the fusion intermediate state, is accessible to molecules less than 70 Kd. Therefore, the antibody is too big (~150 Kd) to access it, while the small molecule compound is too small to block the HR1–HR2 interaction. Moreover, it is safer than small molecule compounds and is more economical than antibodies. The weaknesses of a peptide drug include its short half-life and ability to induce antibodies against the peptide drugs. However, these antiviral peptide drugs are generally used in the early stage of the viral infection for a short period of time (e.g., 2–3 weeks) to save patients’ lives. Therefore, these weaknesses might not significantly affect their clinical use.

## Figures and Tables

**Table 1:** Summary of broad-spectrum coronavirus fusion inhibitors targeting receptor-binding domain (RBD).

Inhibitor Name	Molecular Weight	Target Site	Testing Model	Activity In Vitro (IC_50_)			Activity In Vivo (Protect Rate)	Ref.
				Cell-Cell Fusion	Pseudovirus Infection	Live Virus Infection		
ACE2-Ig	111.38 KD (protein)	ACE2	In vitro 293T/ACE2 cell	0.8 μg/mL (SARS-CoV) 0.1 μg/mL (SARS-CoV-2)	0.85 μg/mL (SARS-CoV) 0.65 μg/mL (SARS-CoV-2)	No reported	No reported	[54]
47D11	150 KD (antibody)	RBD	In vitro Vero E6 cell	20 μg/mL (significantly inhibit, SARS-CoV) 20 μg/mL (significantly inhibit, SARS-CoV-2)	0.06 μg/mL (SARS-CoV) 0.08 μg/mL (SARS-CoV-2)	0.19 μg/mL (SARS-CoV) 0.57 μg/mL (SARS-CoV-2)	No reported	[51]

**Table 2:** Summary of fusion inhibitors targeting fusion-related proteases.

Inhibitor Name	Molecular Weight	Target Site	Testing Model	Activity In Vitro (IC_50_)			Activity In Vivo (Protect Rate)	Ref.
				Cell-Cell Fusion	Pseudovirus Infection	Live Virus Infection		
Camostat mesylate	494.52 (compound)	TMPRSS2	In vitro: Calu-3; 293FT; Vero/TMPRSS2 cell In vivo: BALB/c mice	About 100 nM (MERS-CoV, 293FT cell)	444 nM (MERS-CoV) 198 nM (SARS-CoV) 87 nM (SARS-CoV-2)	About 100 nM (significantly inhibit, MERS-CoV)	~60% (SARS-CoV lethal model)	[60,61,63]
Nafamostat mesylate	539.58 (compound)	TMPRSS2	In vitro: Caclu-3 293FT; Vero/TMPRSS2 cell	100 nM (MERS-CoV, 293FT cell) 1 nM (MERS-CoV, calu-3 cell)	5.9 nM (MERS-CoV) 1.4 nM (SARS-CoV) 5 nM (SARS-CoV-2)	1 nM (significantly inhibit, MERS-CoV)	No reported	[60,61]
Gabexate mesylate	417.48 (compound)	TMPRSS2	In vitro: Calu-3 cell 293FT cell	>1 × 10^5^ nM (MERS-CoV, 293FT cell)	1.15 × 10^5^ nM (SARS-CoV) 1.2 × 10^9^ nM (SARS-CoV-2)	No reported	No reported	[60,61]
K11777	No reported (compound)	CPL	In vitro: 293T/ACE2 293T/CD13 Vero cells Vero 76 cells	No reported	0.68 nM (SARS-CoV) 1.48 nM (HCoV-229E) 6.78 nM (HCoV-NL63) 46.12 nM (MERS-CoV)	<0.05 nM (SARS-CoV)	No reported	[63]
Teicoplanin	1709.39 (compound)	CPL	In vitro: A549 cells HEK293T	No reported	630 nM (MERS-CoV) 3760 nM (SARS-CoV) 1660 nM (SARS-CoV-2)	No reported	No reported	[65,66]

**Table 3:** Summary of the broad-spectrum coronavirus fusion inhibitors targeting the HR1 domain.

Inhibitor Name	Molecular Weight	Target Site	Testing Model	Activity In Vitro (IC_50_)			Activity In Vivo (Protect Rate)	Ref.
				Cell-Cell Fusion	Pseudovirus Infection	Live Virus Infection		
OC43-HR2P	4270.86 (peptide)	HR1	In vitro Huh-7 cells	390 nM (MERS-CoV) 540 nM (SARS-CoV) 660 nM (HCoV-OC43) 840 nM (HCoV-229E) 940 nM (HCoV-NL63)	Similar to EK1 (1810 nM, HCoV-OC43))	930 nM (HCoV-OC43)	No reported	[44]
EK1	4331.98 (peptide)	HR1	In vitro Huh-7 cells 293T/ACE2 cells In vivo Balb/c mice	315 nM (SARS-CoV-2) 180 nM (MERS-CoV) 270 nM (SARS-CoV) 330 nM (HCoV-OC43) 150 nM (HCoV-229E) 630 nM (HCoV-NL63)	2375 nM (SARS-CoV-2) 260 nM (MERS-CoV) 2230 0M (SARS-CoV) 3350 nM (HCoV-229E) 6020 nM (HCoV-NL63) 1810 nM (HCoV-OC43) 2250 nM (Rs 3367) 2010 nM (WIVI)	2468 nM (SARS-CoV-2) 110 nM (MERS-CoV) 620 nM (HCoV-OC43) 690 nM (HCoV-229E) 480 nM (HCoV-NL63)	100% (prophylactic; 0.5 h pre-infection) 66.7% (therapeutic; 0.5 h post-infection)	[34,44]
EK1C4	5436.08 (lipopeptide)	HR1	In vitro Huh-7 cells 293T/ACE2 cells In vivo Balb/c mice	1.3 nM (SARS-CoV-2) 4.3 nM (SARS-CoV) 2.5 nM (MERS-CoV) 7.7 nM (HCoV-OC43) 5.2 nM (HCoV-229E) 21.4 nM (HCoV-NL63) 4.5 nM (WIVI) 8.1 nM (Rs 3367) 4.3 nM (SHC014)	15.8 nM (SARS-CoV-2) 11.7 nM (SARS-CoV) 11.1 nM (MERS-CoV) 37.7 nM (HCoV-OC43) 12.4 nM (HCoV-229E) 76.6 nM (HCoV-NL63) 30.8 nM (WIVI) 66.9 nM (Rs 3367)	36.5 nM (SARS-CoV-2) 4.2 nM (MERS-CoV) 24.8 nM (HCoV-OC43) 101.5 nM (HCoV-229E) 187.6 nM (HCoV-NL63)	100% (prophylactic; 4 h pre-infection) 100% (therapeutic; 0.5 h post- infection)	[34,44]

## Abbreviations

SARS	Severe acute respiratory syndrome
MERS	Middle East respiratory syndrome
COVID-19	Coronavirus Disease 2019
SARS-CoV-2	SARS coronavirus 2
2019-nCoV	Novel coronavirus 2019
HCoV-19	Human coronavirus 2019
SARSr-CoV	SARS-related coronavirus
NTD	N-terminal domain
CTD	C-terminal domain
RBD	Receptor-binding domain
ACE2	Angiotensin-converting enzyme 2
APN	Aminopeptidase N
DPP4	Dipeptidyl peptidase 4
CPL	Cysteine protease cathepsin L
TMPRSS2	Transmembrane protease serine 2
FP	Fusion peptide
6-HB	Six-helix bundle
PRNT	Plaque reduction neutralization test
IFITM	Interferon-inducible transmembrane
NM	Nafamostat mesylate
FOY	Ggabexate mesylate
IC_50_	50% inhibitory concentration
CC_50_	50% cell cytotoxicity concentration
EC_50_	Concentration for 50% of maximal effect
EC_90_	Concentration for 90% of maximal effect
RAS	Renin-angiotensin system
AT2	Angiotensin II receptor
pan-CoV	Pan-coronavirus
